# Encapsulation of the Natural Product Tyrosol in Carbohydrate Nanosystems and Study of Their Binding with ctDNA

**DOI:** 10.3390/polym13010087

**Published:** 2020-12-28

**Authors:** Antonella Rozaria Nefeli Pontillo, Evangelia Konstanteli, Maria M. Bairaktari, Anastasia Detsi

**Affiliations:** 1 Laboratory of Organic Chemistry, Department of Chemical Sciences, School of Chemical Engineering, National Technical University of Athens, 15780 Zografou, Greece; nefelipontillo@gmail.com (A.R.N.P.); e.kwnst@gmail.com (E.K.); maro.bairakt@gmail.com (M.M.B.); 2 Institute of Chemical Biology, National Hellenic Research Foundation, 48 Vassileos Constantinou Avenue, 11635 Athens, Greece

**Keywords:** tyrosol, nanoparticles, Design of Experiment (DoE), chitosan, β cyclodextrin, DNA binding

## Abstract

Tyrosol, a natural product present in olive oil and white wine, possesses a wide range of bioactivity. The aim of this study was to optimize the preparation of nanosystems encapsulating tyrosol in carbohydrate matrices and the investigation of their ability to bind with DNA. The first encapsulation matrix of choice was chitosan using the ionic gelation method. The second matrix was β-cyclodextrin (βCD) using the kneading method. Coating of the tyrosol-βCD ICs with chitosan resulted in a third nanosystem with very interesting properties. Optimal preparation parameters of each nanosystem were obtained through two three-factor, three-level Box-Behnken experimental designs and statistical analysis of the results. Thereafter, the nanoparticles were evaluated for their physical and thermal characteristics using several techniques (DLS, NMR, FT-IR, DSC, TGA). The study was completed with the investigation of the impact of the encapsulation on the ability of tyrosol to bind to calf thymus DNA. The results revealed that tyrosol and all the studied systems bind to the minor groove of ctDNA. Tyrosol interacts with ctDNA via hydrogen bond formation, as predicted via molecular modeling studies and corroborated by the experiments. The tyrosol-chitosan nanosystem does not show any binding to ctDNA whereas the βCD inclusion complex shows analogous interaction with that of free tyrosol.

## 1. Introduction

**Figure 1:**
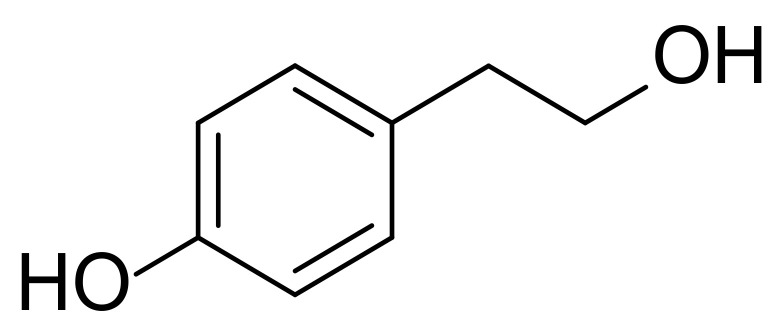
Structure of tyrosol.

Tyrosol (2-(4-Hydroxyphenyl)ethanol) is a biophenol that is found in olive oil, white wine, beer and vermouth (Figure 1) [1]. Even though tyrosol does not exhibit strong antioxidant activity, it contributes to the cellular defences due to intracellular accumulation [2,3]. Moreover, numerous studies affirm that tyrosol offers neuroprotective and cardioprotective effect and enhances the regulation of the human LDL levels [4,5]. However, its hydrophilic nature impedes its incorporation in lipid substrates and limits its absorption and bioavailability [6].

Nanoencapsulation of bioactive compounds and pharmaceutical agents in suitable carriers is a very promising technology, as it offers protection and stabilisation of the encapsulated compound. Furthermore, the encapsulation of a compound may lead to a controllable and sustained release, thus enhancing its activity. Therefore, this technology is incorporated in a broad range of applications in different fields, such as in medicinal and pharmaceutical science, cosmetics, agrochemical and food industry [7,8,9,10].

β-cyclodextrin (βCD) is a truncated cone-shaped oligosaccharide, with a hydrophobic inner cavity and a hydrophilic outer surface [11]. Small, hydrophobic molecules can be entrapped in the cavity forming an inclusion complex (IC), increasing their solubility, while more hydrophilic compounds can be bound on the external surface [12,13,14].

Chitosan (CS) is a naturally occurring polymer widely used as a nanocarrier. It is nontoxic, biocompatible and biodegradable and is recognised as Generally Recognised as Safe (GRAS) by the Food and Drug Administration (FDA) [15,16]. The process of encapsulation in chitosan nanoparticles (NPs) has been extensively studied and various techniques have been reported. The nature of the polymer permits the encapsulation of small or larger molecules, natural products like plant extracts and essential oils, or even other nanosystems [17,18,19].

The properties of the particulate system are defined by the selected carrier and preparation process. Therefore, the ability to design and engineer the experimental process in order to obtain desirable results is an asset for any application. To that end, experimental design and statistical analysis are implemented. Box-Behnken design (BBD) is a Response Surface Methodology (RSM) that enables the multivariate optimisation of a quadratic model [20,21,22].

Intercalators and groove binders are a class of compounds that interact with the double-stranded DNA. Many anticancer drugs, such as anthracyclines, interact with the DNA through intercalation between adjacent base pairs perpendicularly to the axis of the helix. Many substituents in the intercalator molecule can greatly influence the binding mechanism, the geometry of the ligand–DNA complex and the selectivity of the sequence [23,24].

The interactions between the various cyclodextrins and DNA have yet to be completely identified; however, they are of utmost importance as there are many marketed formulations that contain cyclodextrins. Modified cationic cyclodextrins are known to interact with DNA for gene therapy applications while a strong interaction of a βCD complex was proved to be formed with DNA as the ribose and phosphate groups of the DNA exert a stabilizing effect by forming H-bonds with the outer surface of CD [25,26].

The aim of this study was to develop and optimize the encapsulation process of tyrosol in nanosystems using different matrices namely: chitosan (**TYR/CS**), βCD (**TYR-βCD**) as well as in the combined system of βCD/CS (**TYR-βCD/CS**). The kneading method was used for the preparation of the inclusion complex of tyrosol with βCD (**TYR-βCD**) and ionic gelation for the synthesis of the chitosan nanoparticles. The process optimisation was performed in both cases using a three-factor three-level BBD. The independent variables were set as the initial concentration of the polymer, the loading capacity of **TYR** or the **TYR-βCD** inclusion complex and the amount of the cross-linking agent. The examined range was elicited from literature data and data from preliminary experiments.

Complete characterisation of the systems was performed by various methods and techniques, such as Nuclear Magnetic Resonance Spectroscopy (NMR), Infrared Spectroscopy (FT-IR), antioxidant activity determination by the DPPH method, Dynamic Light Scattering (DLS) for the measurement of size, polydispersity index and ζ-potential, Thermogravimetric Analysis (TGA) and Scanning Electron Microscopy (SEM). Finally, the effect of the encapsulation matrix on the ability of tyrosol to interact with Calf-thymus DNA (ctDNA) was investigated.

## 2. Materials and Methods

### 2.1. Materials

Tyrosol was purchased from Fluorochem (Hadfield, Derbyshire, UK), β-Cyclodextrin in an assay ≥99% (HPLC) was obtained from Fluka (Buchs, Switzerland) and Chitosan (5–20 mPa·s, 0.5% in 0.5% Acetic Acid at 20 °C) from TCI (TCI (Shanghai, China). Tween 80, Tris Base, rhodamine B and deoxyribonucleic acid sodium salt, calf thymus were purchased from Alfa Aesar (Ward Hill, MA, USA) and Sodium Tripolyphosphate from Acros Organics (Morris Plains, NJ, USA). For all the experiments double-deionised water was used.

### 2.2. Synthesis of Nanoparticles

#### 2.2.1. Preparation of Inclusion Complexes of Tyrosol with βCD (**TYR-βCD**)

The kneading method was used for the preparation of the inclusion complex. Briefly, equimolar quantities of βCD (569 mg) and TYR (70 mg) were mixed in an agate mortar and pestle with minimum amount of the solvent (H_2_O:EtOH 7:3 v/v) for 30 min. The paste was dried to constant weight in a high vacuum pump [27].

#### 2.2.2. Encapsulation of Tyrosol in Chitosan Nanoparticles (**TYR/CS**)

In a 1% aqueous acetic acid solution, 0.05%, 0.2% or 0.35% CS was fully dissolved. Then, an equal to CS amount of emulsifier Tween 80 is added and left to stir at room temperature overnight until complete dissolution. In 5 mL of the occurring solution 2.5, 10 or 17.5 mg of tyrosol was added and the solution was left to stir until total dissolution. Then 1 mL of TPP solution of concentration 0.42, 1.67 or 0.35 mg/mL was added dropwise. The sample was left under magnetic stirring at 700 rpm, for 10 min at 25 °C. The nanoparticles were centrifuged at 30,000 rpm for 45 min. The sediment was dispersed and washed with two more consecutive centrifugations. Finally, the nanoparticles were freeze dried and stored in a desiccator.

#### 2.2.3. Coating of the Tyrosol-βCD Inclusion Complexes with Chitosan (**TYR-βCD/CS**)

For the synthesis of **TYR-βCD/CS** nanoparticles, the procedure described in “Section 2.2.2” was followed, adding **TYR-βCD** instead of tyrosol. For preparation of blank chitosan nanoparticles, in 5 mL of 0.2% chitosan, 1 mL of TPP solution 1.67 mg/mL was added and the solution was left to stir for 10 min.

### 2.3. Design of Experiments (DoE)

Two experimental designs were conducted to optimize the processes for preparation of **TYR/CS** and **TYR-βCD/CS** nanoparticles. Design-Expert^®^ in trial version (Version 12, Stat-Ease, Inc., Minneapolis, MN, USA) was used.

Three factors at three levels were selected to control the size (response R_1_) and ζ-potential (response R_2_) of the nanosystem. Factor A was the concentration of the chitosan solution, factor B was the mg of TPP and factor **C** the amount of tyrosol for the **TYR/CS** nanosystem or of the inclusion complex for the **TYR-βCD/CS** system.

Factors’ levels and their normalised values are shown in Table 1. Central Point (0, 0, 0) was repeated three times, and a total of 15 runs were performed for each set.

The data obtained were analysed with Analysis of Variance (ANOVA). Linear, second-order and quadratic models were evaluated for all responses and in terms of statistical significance, R^2^ values and the deviation of the predicted to the experimentally obtained results.

The confidence level was set at 95% and *p*-values ≤ 0.05 to determine statistically significant factors.

### 2.4. Characterisation of the Nanoparticles

#### 2.4.1. Dynamic Light Scattering (DLS)

The measurements for size, polydispersity index (PDI) and ζ-potential were performed in a Zetasizer Nano ZS, Malvern Instruments Ltd. (Malvern, UK) using a cuvette DTS1070. The results were analysed with the Zetasizer 7.12, Malvern Instruments Ltd. Software.

For **TYR-βCD**, 1 mg of the dried sample was dissolved in 4 mL of water and was fully dispersed with 2 min ultrasound at a 2210 Ultrasonic Bath, Branson. DLS measurements of the **TYR/CS and TYR-βCD/CS** samples were conducted by diluting 1 mL of freshly made sample in 1 mL of water.

#### 2.4.2. Encapsulation Efficiency (EE%) and Loading Capacity (LC%)

After ultracentrifugation, the quantification of free tyrosol in the supernatant was performed using UV-Vis spectroscopy.

(1) $$EE\%=\;\frac{Total\;TYR\;\left(mg\right)-TYR\;in\;supernatant\;\left(mg\right)}{Total\;TYR\;\left(mg\right)}\times 100$$

*LC% = [Total encapsulated (mg)/total nanoparticles weight (mg)] × 100* (2)

#### 2.4.3. Release Study

The release profile of tyrosol was investigated by determining the quantity of tyrosol released form the nanosystem at given time intervals. For that reason, 50 mg of the each nanosystem were dissolved in a 12 mL solution of aqueous 1% acetic acid (6 mL) and 6 mL DMSO and were stirred at 37 °C at 100 rpm. At specific time intervals, 0.5 mL of sample was obtained and filtered through a 0.45 μm pore syringe filter and analysed by UV-Vis spectroscopy. Each time 0.5 mL of fresh solvent was added to the solution

#### 2.4.4. Fourier Transform Infrared Spectroscopy (FTIR)

KBr pellets containing the dried sample were prepared with hydraulic pellet press. The FT-IR spectra were recorded with a JASCO FT/IR-4200 spectrometer (Japan Spectroscopic Company, Tokyo, Japan).

#### 2.4.5. Nuclear Magnetic Resonance Spectroscopy (NMR)

^1^H NMR spectrum of TYR-βCD IC was obtained to determine the host–guest interactions. The spectra were obtained on a Varian V 600 MHz instrument (National Hellenic Research Foundation, Institute of Chemical Biology, Athens, Greece). The inclusion complex was dissolved in deuterium oxide (D_2_O).

#### 2.4.6. Thermal Characterisation

Thermal characterisation of the dried samples was performed with Differential Scanning Calorimetry (DSC) with a DSC 1 STARe System device (Mettler Toledo, Columbus, OH, USA) at temperature range from 20 °C to 350 °C with heating rate 10 °C/min, under nitrogen gas flow 20 mL/min and Thermogravimetric Analysis (TGA) the TGA/DSC 1 STARe System Thermobalance (Mettler Toledo, Columbus, OH, USA) at 25 °C–600 °C, with heating rate 10 °C/min, under nitrogen gas flow 10 mL/min.

### 2.5. Molecular Docking

The study of the interaction mode and binding affinity docking studies has been performed with the crystal structure of the DNA (PDB ID: 1bna), was obtained from the RSCB protein Data Bank. The optimisation of the docking parameters was performed using AutoDock Vina software (The Scripps Research Institute, La Jolla, CA, USA) and implemented empirical free energy function. Only polar hydrogens were added to the DNA in AutoDock Tools [28]. Finally, the image has been generated using PyMol software. The name and the number of nucleotides were designed according to PyMol software.

### 2.6. DNA Binding Studies Using UV-Vis Spectroscopy

Lyophilised Calf-thymus DNA (ctDNA) was dissolved in Tris-HCl buffer solution of concentration 10 mM and pH 7.4, and left overnight at 4 °C. Then, 1 mg ctDNA was dissolved in 1 mL buffer and the concentration was determined from the absorbance at 260 nm using an extinction coefficient of 6600 M^−1^ cm^−1^ [29]. Tyrosol, βCD, CS, TYR-βCD, TYR-βCD/CS, TYR/CS and Rhodamine B were dissolved in the buffer to a concentration of 10 mM for tyrosol or Rhodamine B or 2 mg/mL for the two carriers and all the nanosystems, which were then used as the stock solution for the preparation of the concentration of 100μΜ. Afterwards, various concentrations (0–100 μM) of ctDNA were added to the prepared solutions which were incubated for 5 min and 30 min at 37 °C. Absorption spectra were measured using a JASCO double beam V-770 UV-Vis/NIR spectrophotometer in range of 230–400 nm.

## 3. Results

### 3.1. DoE for the TYR/CS Preparation Process

The measured responses for the **TYR/CS** nanoparticles are presented in Table 2.

From the table above, it can be observed that the size of the occurring nanoparticles ranged from 115.3 nm at central point to 679.6 nm except for points (−1, 1, 0), (−1, 0, 1) and (−1, 0, −1) in which the particles were over 3 μm and precipitated.

ζ-potential was positive in all cases and ranged from 4.3 to 46.4 mV. As chitosan is a cationic polymer at acidic environments, highly positive ζ-potential is expected, and is indicative of its stability in dispersion. However, the presence of the polyanion TPP manages to reduce the value, by interacting with the protonated amino groups.

Particles in the micro scale were observed only in three points in all of which the concentration of chitosan was at the low level while the concentration of TPP was on its medium or high level. Hence, it can be deduced that when the ratio of chitosan to TPP is low, there is accumulation of the cross-linker in the particles surface, which can also be confirmed by the low ζ-potential. On the other hand, increase in the particles’ size is also observed when the ratio of chitosan to TPP is high, as it occurs from points (1, −1, 0), (0, −1, −1) and (0, −1, 1). This could be attributed to an excess of chitosan in the particles surface resulting in insufficient cross-linking of the polymer and low cross-linking density between the polymer and the cross-linking agent. The high ζ-potential of those points confirms the existence of protonated amino groups on the surface of the NPs. Moreover, increased concentration of chitosan leads in decreased intermolecular distance and increased intermolecular hydrogen bonding between the polymeric chains [30,31,32,33,34].

**Figure 2:**
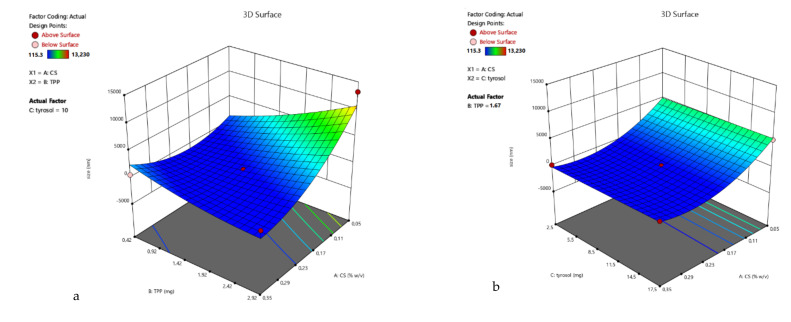

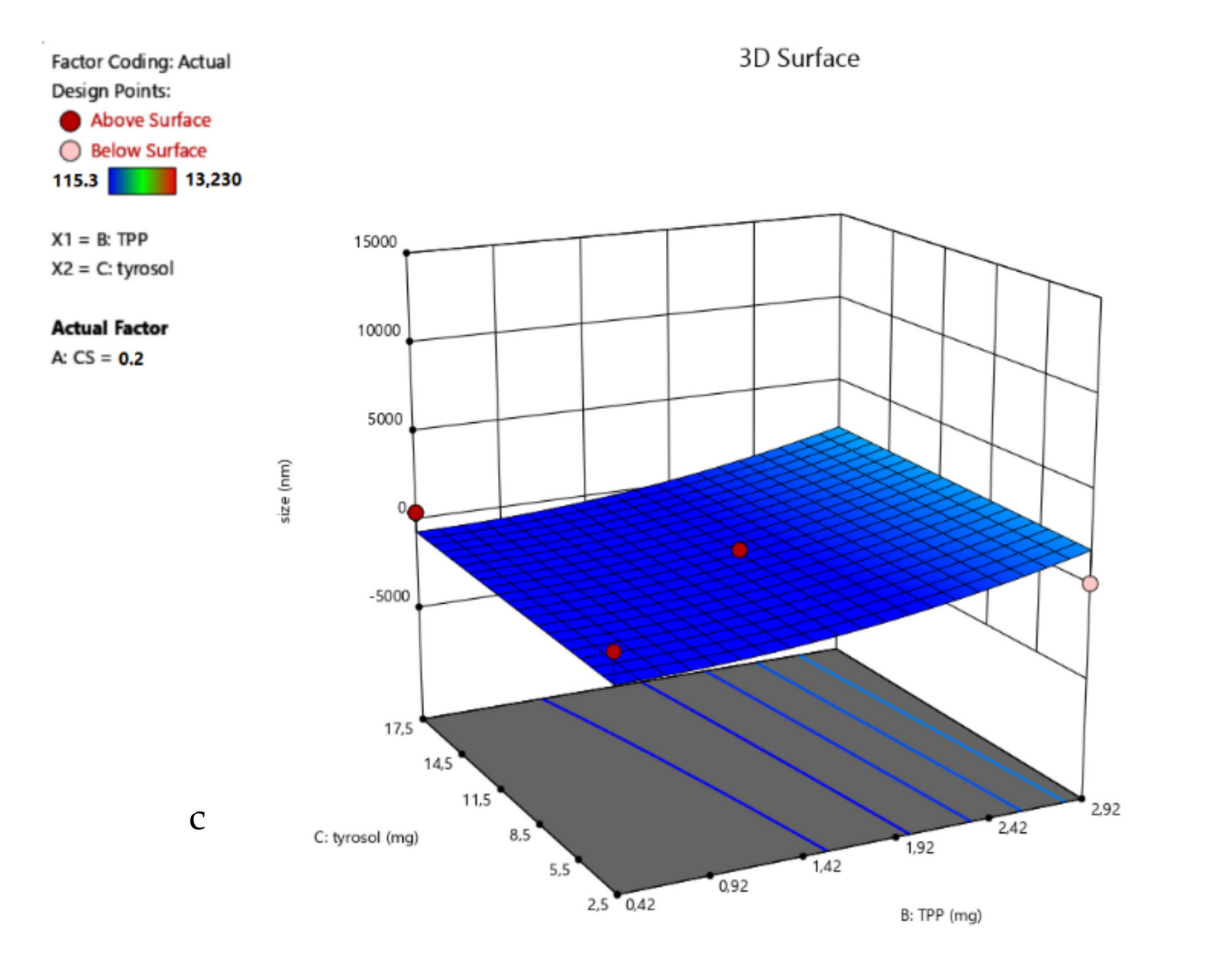
3D surface plot of response R1 of TYR/CS system (**a**) CS Vs TPP (tyrosol: 10 mg) (**b**) CS Vs TYR (TPP: 1.67 mg) (**c**) TPP Vs TYR (CS: 0.2%).

3D surface plots of R_1_ were designed (Figure 2), and statistical analysis of the results was performed. A Reduced Quadratic Model better described the results (Equation (3)) and was found to be statistically significant (*p*-value 0.0012). Factors A, B, AB, A^2^ were found to be statistically significant, verifying the observation that the size of the nanoparticles depends both on the amount of chitosan and the interaction with the crosslinking agent TPP. The Model F-value was calculated to be 9.43, indicating a significant model.

The coded equation that describes the size response is:

R_1_= −85.74 − 2666.41 A + 1450.25 B + 162.06 C − 3301.28 AB + 2674.75 A^2^ + 729.22 B^2^ (3)

**Figure 3:**
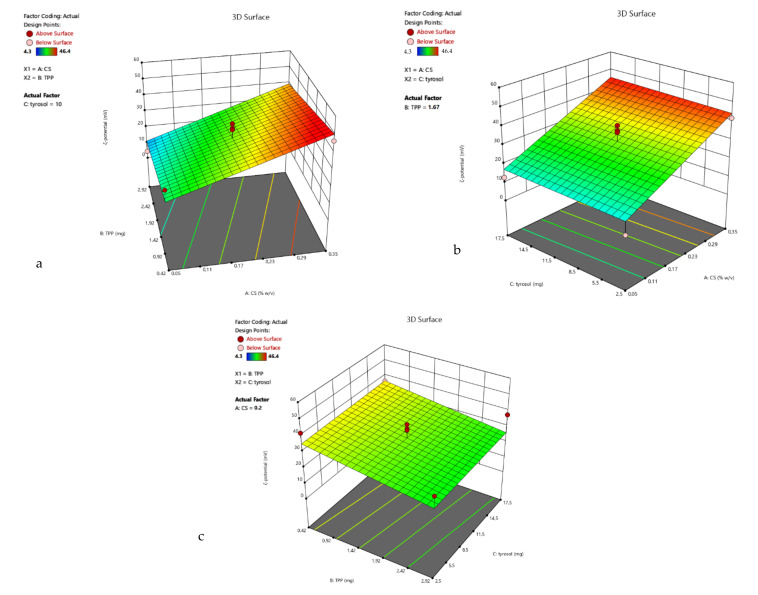
3D surface plot of response R_2_ of TYR/CS system (**a**) CS Vs TPP (tyrosol: 10 mg) (**b**) CS Vs TYR (TPP: 1.67 mg) (**c**) TPP Vs TYR (CS: 0.2%).

For the ζ-potential response, the linear model (Equation (4)) best described the relation between the experiment data of R_2_ and the model F-value was 9.64. The 3D surface plots of R_2_ are presented in Figure 3.

The coded equation that describes the ζ-potential-response is:

R_2_ = 30.64 + 14.25 A − 5.29 B + 0.74 C (4)

Table 3 summarises the significance of each factor for the responses R_1_ and R_2_ of the TYR/CS nanosystem.

Optimal preparation conditions for TYR/CS nanoparticles are found to be close to the Central Point (CP) values of factors B and C and in the range 0.2–0.35% for the CS concentration.

Those results are in accordance with literature. Shah et al. [31], prepared CS nanoparticles loaded with quetiapine fumarate sized between 140 and 487 nm. The optimal preparation conditions were CS concentration 0.1% and CS:TPP ratio 4.8:1, with stirring time 15 min at 700 rpm and resulted in nanoparticles of size 131.08 nm with ζ-potential 34.4 mV. Delan et al. [30] used BBD for the optimisation of the synthesis of chitosan nanoparticles loaded with the anionic and lipophilic drug simvastatin. It was found that the best CS concentration was 0.34% and the CS:TPP ratio was 3:1, leading to nanoparticles of size 106 nm and ζ-potential 43.3 mv. Sharma et al. [34] ran a four-factor, three-level BBD to assess the process of synthesis of Carvedilol loaded CS nanoparticles. In their research, optimum CS concentration was found to be 0.262% and the nanoparticle size was measured with TEM 102.12 nm.

### 3.2. DoE for TYR-βCD/CS

The aqueous dispersion of the inclusion complex of tyrosol with βCD is formed by nanoparticles of size 478.1 nm with a negative, almost neutral ζ-potential of −7.18 mV. The entrapment of the inclusion complex into the chains of chitosan reversed the ζ-potential, due to the presence of chitosan in the outer layer of the inclusion complexes. Furthermore, in most of the 15 runs of the experimental design, the obtained particles were significantly smaller than the inclusion complex. This could be attributed to the strong electrostatic interaction between the oppositely charged carriers, which could separate agglomerations.

From the data in Table 4, it can be observed that the measured sizes ranged from 132.6 nm to over 3 μm, which precipitate, while the ζ-potential ranged from 4.8 to 46.5 mV.

**Figure 4:**
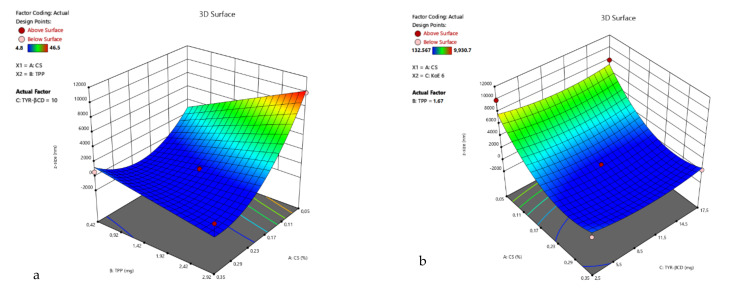

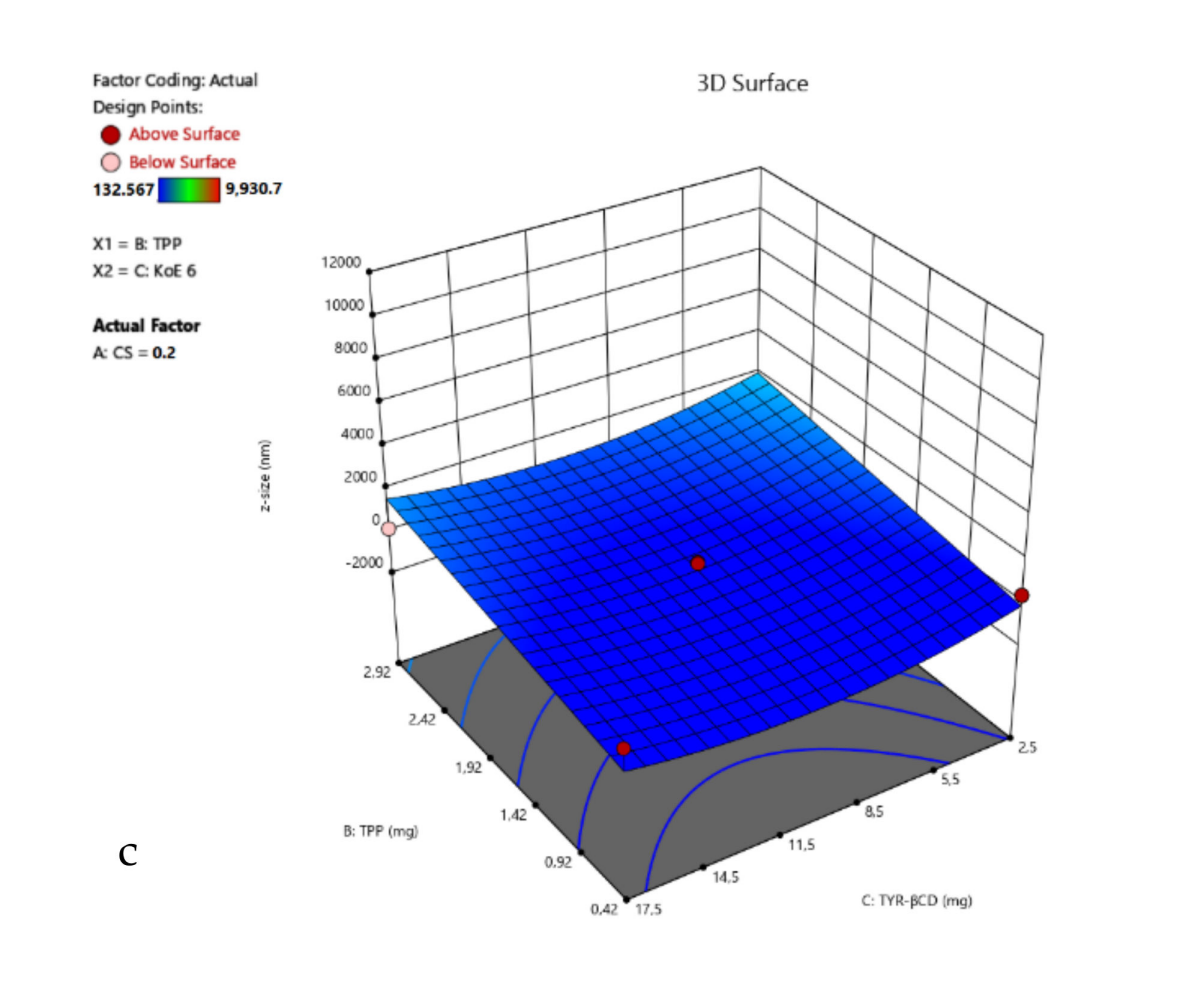
3D surface plot of response R_1_ of TYR-βCD/CS system (**a**) CS Vs TPP (TYR-βCD: 10 mg) (**b**) CS Vs TYR-βCD (TPP: 1.67 mg) (**c**) TPP Vs TYR-βCD (CS: 0.2%).

The lowest result for R_1_ is at the point (0, +1, −1), but at the CP, the size is also very close (average is 190.5 nm). In this design, it can also be observed that the chitosan-to-TPP ratio has a strong impact on the particles’ size and ζ-potential. Therefore, for points (−1, 1, 0), (−1, 0, −1) and (−1, 0, 1), the particles precipitate, and the ζ-potential is very low. Moreover, from the 3D surface plot of the response R_1_ (Figure 4), the size tends to decrease when the concentration of CS increases.

According to the mathematical analysis, the Reduced Quadratic Model was the most suitable for describing this response (F = 12.08). The coded equation (Equation (5)) that describes the system is:

R_1_ = −185.97 − 3350.14 A + 1093.59 B − 226.53 C − 2506.92 AB + 3392.22 A^2^ + 906.57 C^2^, (5)

The ζ-potential values range from 4.8 to 46.5 mV for the TYR-βCD/CS system.

Response R_2_ is best described by the quadratic model, with F-value = 24.02. The coded equation (Equation (6)) of the model is as follows:

R_2_ = 25.04 + 13.11 A − 4.30 B + 2.54 C + 2.18 AB + 1.50 AC − 1.03 BC − 6.72 A^2^ + 7.21 B^2^ + 5.98 C^2^, (6)

**Figure 5:**
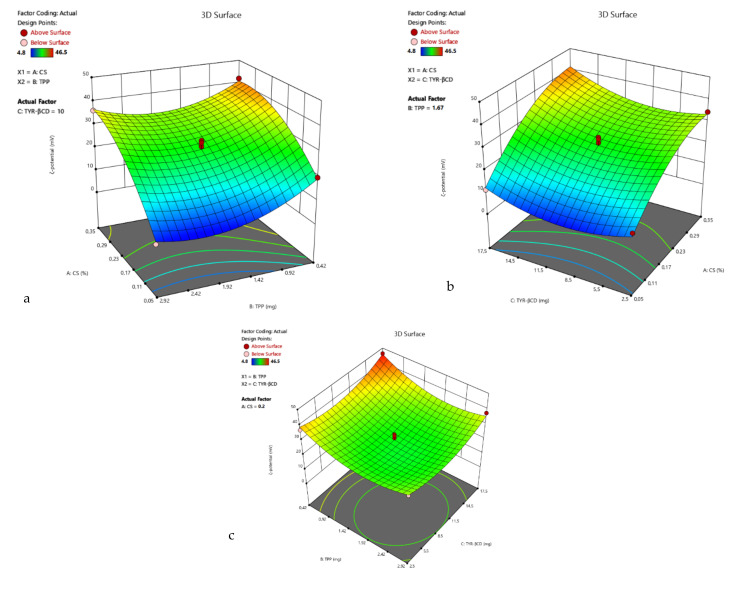
3D surface plot of response R_2_ of the TYR-βCD/CS system (**a**) CS Vs TPP (TYR-βCD: 10 mg) (**b**) CS Vs TYR-βCD (TPP: 1.67 mg) (**c**) TPP Vs TYR-βCD (CS: 0.2%).

The 3D surface plots of R_2_ are presented in Figure 5.

Table 5 summarises the significance of each factor for the responses R_1_ and R_2_ of the **TYR-βCD/CS** nanosystem.

For this system, the optimal preparation formula was found to be the CP, giving the smallest nanoparticles and a ζ-potential of 25.9 mV. This point was chosen for further experiments.

Comparing the experimental results, the lowest size values were given at the point (0, +1, −1) and the CP. In both cases, the ζ-potential was highly positive; hence, the central point was chosen for comparative reasons. This result is in accordance with the software’s predictions of the optimal points.

Comparing the two nanosystems, many similarities can be observed. First, the initial concentration of chitosan in the nanoparticle-forming solution plays a significant role in the properties of the particles. Increased concentration of chitosan results in the agglomeration of particles and a very high ζ-potential, attributed to the presence of many protonated amino groups. On the other hand, chitosan to TPP ratio is also important and strongly affects both responses. Moreover, the range of both responses does not differ significantly for the two systems, suggesting that the polymeric chains form a matrix entrapping the molecule or the inclusion complex and form the nanoparticles.

The main difference between the two systems is the impact of the **TYR-βCD** to the ζ-potential. The reason for this difference could be that the inclusion complex is negatively charged and that a strong electrostatic interaction between the oligosaccharide and chitosan exists.

### 3.3. Encapsulation Efficiency and Loading Capacity Calculation

After identifying the optimal conditions for obtaining nanoparticles, the encapsulation efficiency (EE%) and loading capacities (LC%) were determined for the three nanosystems.

**Figure 6:**
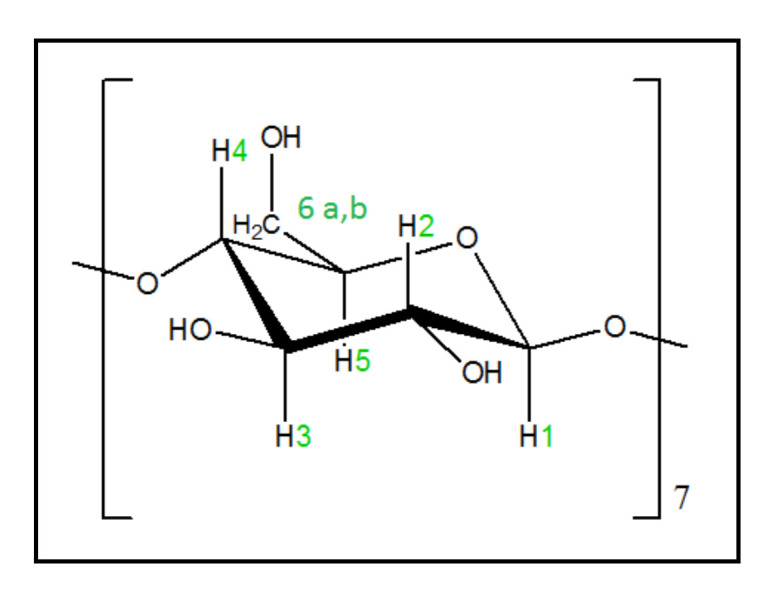
The glucose monomer in βCD.

For the inclusion complex of tyrosol with the βCD, it was found that the EE% was 98%. The high encapsulation efficiency is expected for this system, as the kneading technique was implemented, and no washing of the tyrosol was performed. The structure of βCd is presented in Figure 6.

For the **TYR/CS** nanosystem, the EE% was found to be 46% and the LC 12%, while for the **TYR-βCD/CS** nanosystem, the corresponding values were 12 and 4.2%, respectively.

### 3.4. Structural Identification of **TYR-βCD** Using ^1^H NMR Spectroscopy

**Figure 7:**
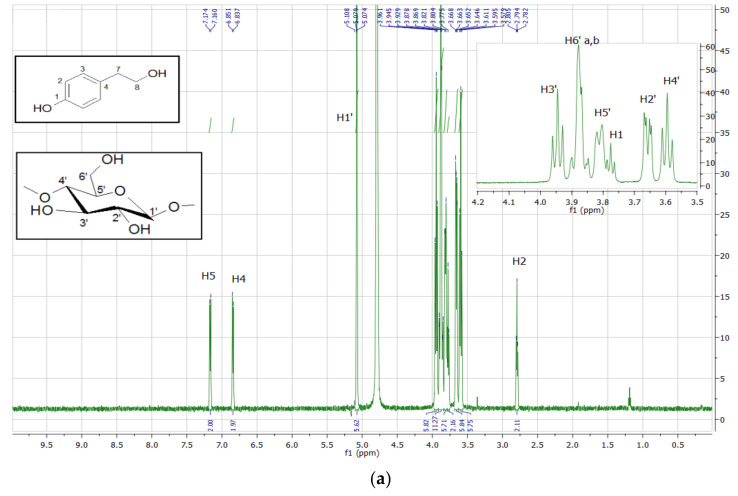

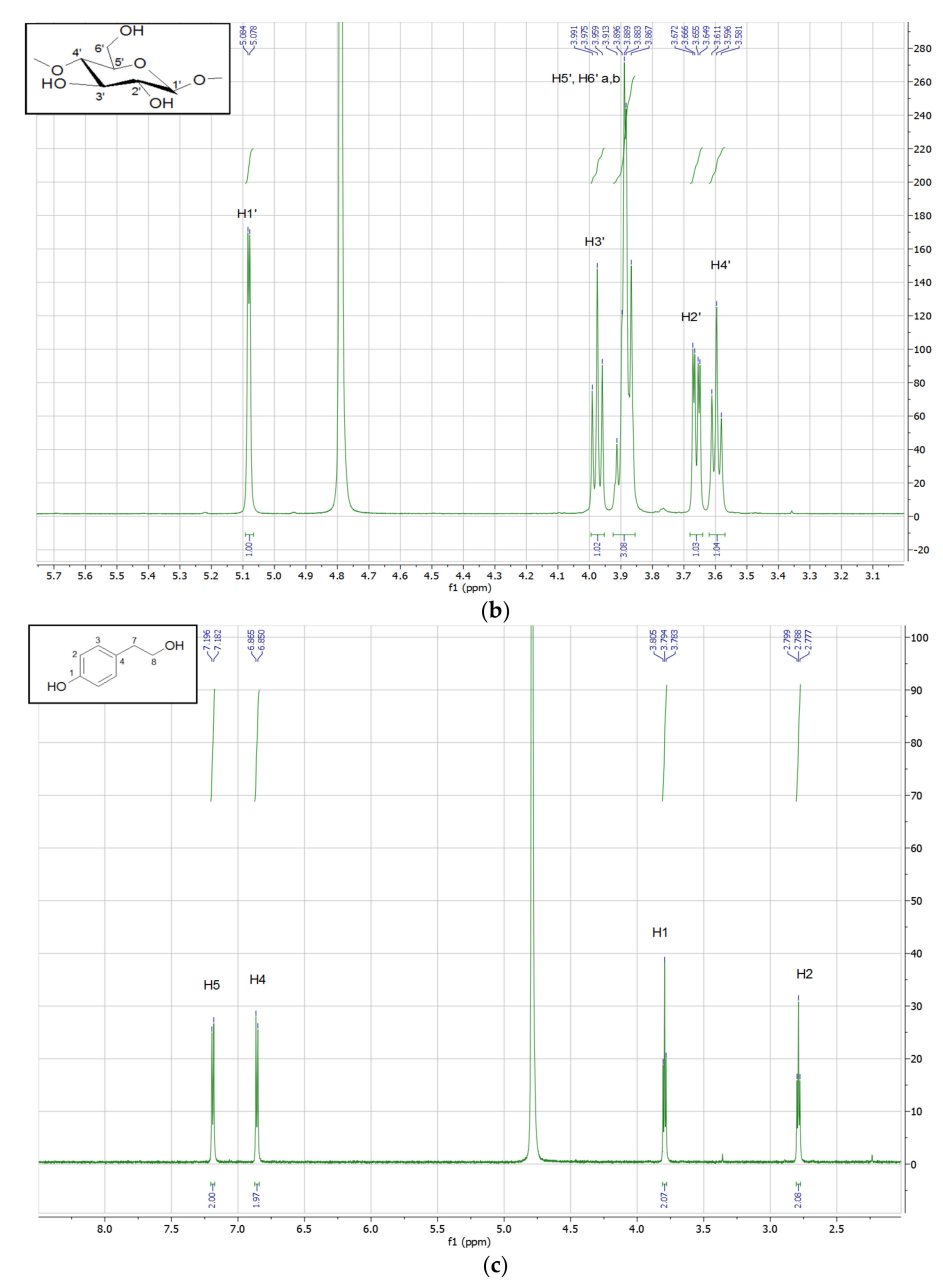
^1^H NMR spectrum (600MHz, D_2_O) of (**a**) **TYR-βCD** and expansion of the region 3.4–4.1 ppm, (**b**) βCD, and (**c**) tyrosol.

The analysis of the ^1^H NMR spectrum of the inclusion complexes with βCD provides important evidence regarding the host–guest interactions. The ^1^H NMR spectrum of **TYR-βCD,** as well as those of tyrosol and βCD, are presented in Figure 7. In Table 6, the chemical shift changes of ^1^H-NMR signals of the protons of βCD before and after the formation of the **TYR-βCD** inclusion complexes are shown.

The peaks of the H-3 and H-5 protons of βCD (which are located inside the cavity) present significant upfield shift (−0.030 and −0.100 ppm, respectively), indicative of strong hydrophobic interactions between tyrosol and βCD inside the cavity. The upfield shift of the H-6 (−0.039 ppm), which lie at the primary face of the cyclodextrin cone, implies that there is also a strong interaction with tyrosol, probably between the aliphatic OH of the tyrosol molecule and the 6-OH of βCD. These results lead us to believe that the tyrosol molecule enters the cyclodextrin cone in such a way that the aromatic ring lies well inside the hydrophobic cavity whereas the aliphatic hydroxyethyl moiety points towards the primary face of the cone and strong hydrogen bonds are formed between the OH groups. These observations are in accordance with the results of Rescifina et al. [35] and Lopez-Garcia et al. [36], who studied the structure of the inclusion complexes of tyrosol and hydroxytyrosol with βCD.

### 3.5. FT-IR Analysis of the TYR-β-CD IC

**Figure 8:**
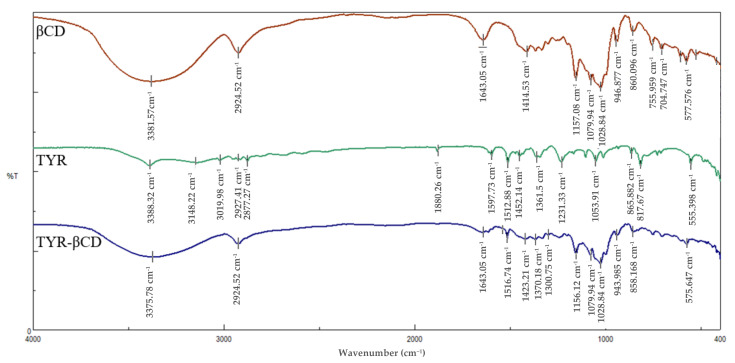
FT-IR spectra of β-CD (red), tyrosol (green) and tyrosol-β-CD inclusion complex (blue).

The analysis of the FT-IR spectra of pure βCD, tyrosol and the inclusion complex can serve as further proof of the formation of the inclusion complex. The most characteristic peaks in the FT-IR spectra of the above-mentioned compounds are shown in Table 7 and the spectra in Figure 8.

The FT-IR spectrum of the inclusion complex of tyrosol with βCD (TYR-βCD) shows a broad absorption band at 3376 cm^−1^ owed to the OH stretching vibration. This band is shifted compared to the corresponding band at the spectra of pure βCD (3382 cm^−1^) and tyrosol (3389 cm^−1^). This shift is indicative of strong interactive forces between the host and guest in the inclusion complex. The band at 1543 cm^−1^ present in the FT-IR spectrum of the inclusion complex is attributed to the C=C stretching vibration in aromatic compounds and is shifted by 29 cm^−1^ from the value of the same band at the spectrum of pure tyrosol. This large shift is further evidence of the efficient inclusion of tyrosol in the cyclodextrin cone with the aromatic part of the molecule lying inside the cone, as concluded also by the NMR spectra. The absorption band of the OH bending vibration appears at 1423 cm^−1^ in the spectrum of the inclusion complex and is shifted from the value of the same absorbance at the spectra of β-CD and tyrosol by 9 cm^−1^ and 29 cm^−1^, respectively. Again, these shifts corroborate the strong interaction between tyrosol and β-CD.

### 3.6. Differential Scanning Calorimetry Analysis (DSC)

**Figure 9:**
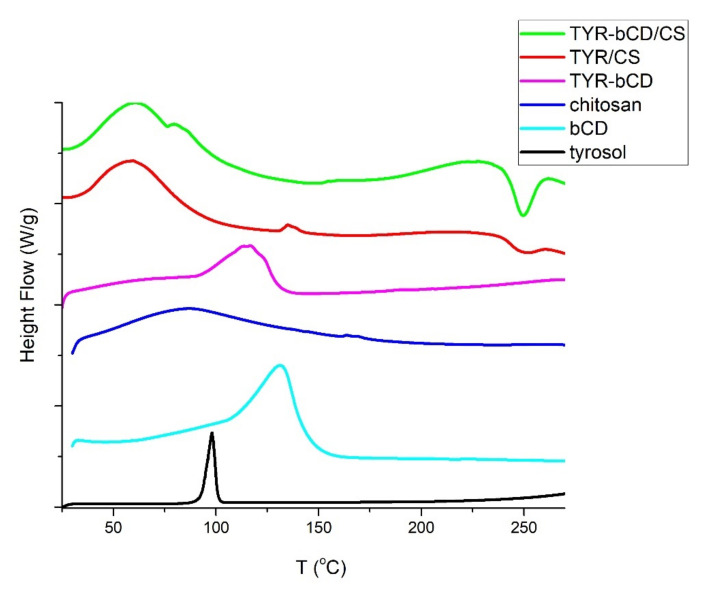
DSC thermograms of tyrosol (black), β-cyclodextrin (cyan), chitosan (blue), TYR-βCD inclusion complex (magenta), TYR/CS (red), TYR-βCD/CS (green).

The DSC thermograms of tyrosol, the two carbohydrate carriers and the corresponding nanosystems are presented in Figure 9.

In the DSC thermogram of tyrosol, the sharp endothermic process at 85–113 °C with peak at 95 °C, corresponds to the melting point of the compound [6]. In the chitosan DSC thermogram, in the studied range, only the loss of water is observed at the temperature range 85–107 °C, with a peak at 94 °C corresponding to the loss of water. The loss of water from cyclodextrin occurs in the range 98–155 °C with an endothermic peak at 131 °C [37,38,39].

The DSC thermogram of the inclusion complex undergoes an endothermic thermal transition from 87 to 133 °C with peak observed at 116 °C, ascribed to the water loss. The melting of tyrosol cannot be observed in this curve, yet the decrease of the temperature of water loss, compared to the one of the pure βCD, may be evidence of the protection that the carrier offers to the molecule. This is in accordance with what has previously been reported when encapsulating a molecule in βCD [37,39].

In the **TYR/CS** nanosystem, water loss occurs at 60 °C. Similarly, in the **TYR-βCD/CS** nanosystem, the water loss is observed with an endothermic peak at 61 °C. Moreover, in this system a second endothermic process takes place at the temperature range 75–87 °C with peak at 79 °C and could correspond to the loss of the water bound inside the cavity of the βCD.

The endothermic peak owed to the melting of tyrosol is not present in any of the nanosystems studied, and can be considered as further evidence of the successful encapsulation of the compound in the different matrices.

### 3.7. Thermogravimetric Analysis (TGA)

**Figure 10:**
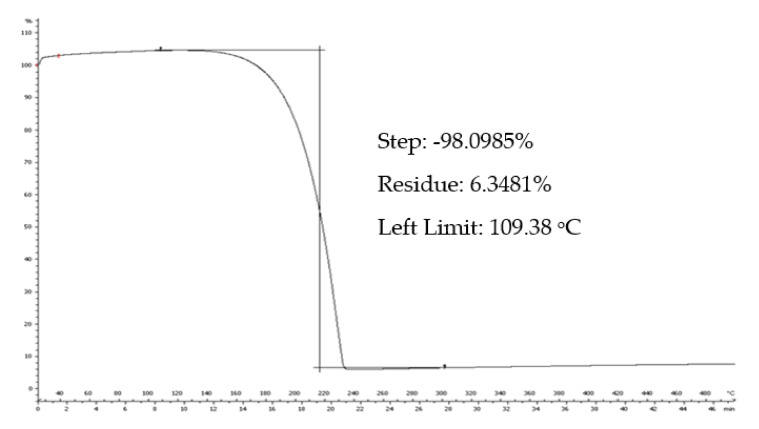
The TGA thermogram of tyrosol.

The thermal stability of the samples can be determined using TGA. As can be seen in Figure 10, the degradation temperature (T_d_) of TYR is 220 °C.

**Figure 11:**
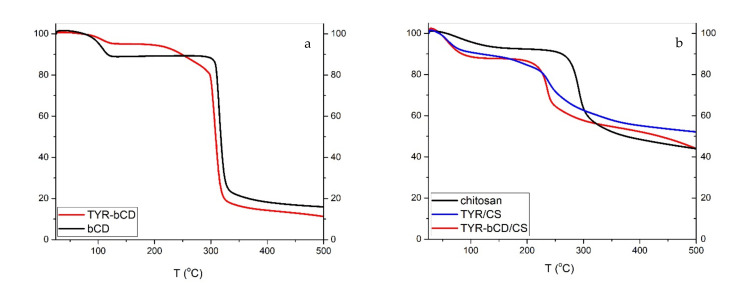
TGA thermograms of (**a**) β-cyclodextrin and TYR-βCD and (**b**) chitosan, TYR/CS and TYR-βCD.

The thermal degradation of chitosan (Figure 11) occurs in two stages: water loss occurs at 92 °C, resulting in an 8% mass loss; whereas the decomposition temperature is 300 °C. The residue of the polymer at 500 °C is approximately 44%.

For the **TYR/CS** nanosystem, the TGA profile presents three stages of degradation ((Figure 11). First, there is the water loss of at 67 °C then there is a mass loss of 5.0% at 187 °C that could be ascribed to tyrosol and, finally, the decomposition of chitosan occurs at 270 °C, leaving a residue of 52% at 500 °C. The slight decrease in the T_d_ of chitosan is attributed to the formation of nanoparticles through anionic gelation and, hence, the synthesis of a new material [40].

In the TG curve of βCD, first there is the loss of water molecules, externally and internally bound, at 103 °C, resulting in a decrease of 11% of the total mass [41]. The decomposition of βCD happens at 323.8 °C and the mass loss is 72.4%. The degradation profile of **TYR-βCD** presents three stages: the water loss at 105 °C with 4.7% mass loss, then the decomposition of tyrosol at 266 °C, resulting in further mass loss of 9.5%, and finally, at 316 °C, the decomposition of the βCD. The decomposition of tyrosol happens at a significantly higher temperature compared to the decomposition of the free molecule, confirming that the formation of the inclusion complex protects it from thermal degradation. The residue of the inclusion complex at 500 °C is 11%.

For the double-encapsulation system **TYR-βCD/CS**, TGA reveals an improved thermal stability of the system. This system degrades in two stages: at 64 °C, the dehydration of the sample happens, losing approximately 14% of the mass, and at the temperature range 161–438 °C, there is another mass loss of 37%, which corresponds to the degradation of the nanosystem. The residue of the sample at 500 °C is 44%.

Therefore, it seems that tyrosol is better protected in the double encapsulated system than in the chitosan matrix.

### 3.8. Molecular Docking

**Figure 12:**
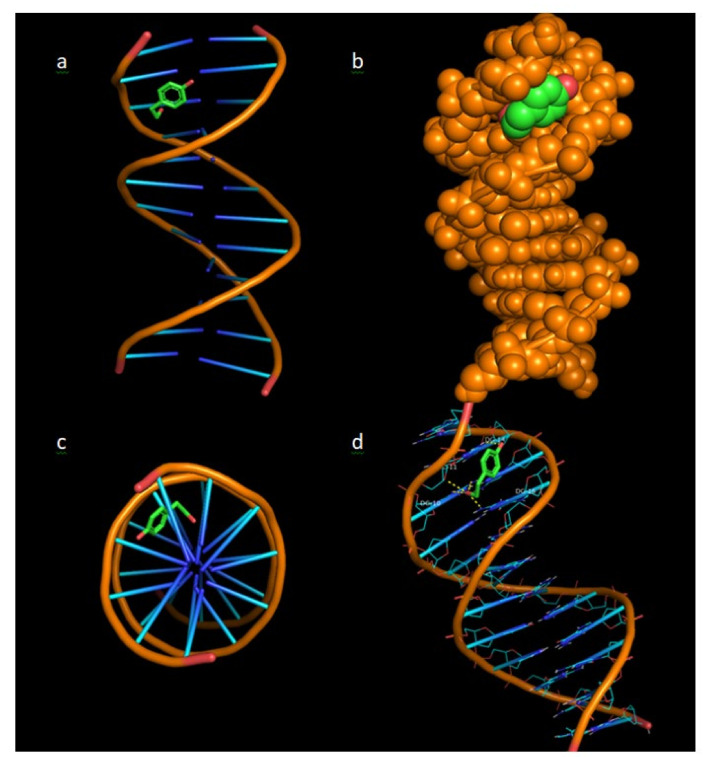
Binding architecture of tyrosol in the crystal structure of CT DNA (PDB:1bna) depicting its stabilisation in the binding cavity of minor groove of DNA. (**a**) DNA structure and tyrosol are illustrated as cartoons, (**b**) DNA structure and tyrosol are formed as spheres, (**c**) The docking pose from a view above the axis of the helix, (**d**) Nucleotides are rendered in line mode and the yellow dotted lines indicate hydrogen bonds between the docked molecule and the nucleotides of the binding pocket in the minor groove of DNA.

In Figure 12, the binding architecture of tyrosol in the crystal structure of DNA (source: PDB:1bna) is presented, depicting its stabilisation in the binding cavity of minor groove of DNA. The docked complex between tyrosol and DNA is illustrated as cartoon (Figure 12a,c,d) and in the form of spheres (Figure 12b) showing the interaction of tyrosol in the binding cavity of minor groove of DNA. The minor groove is smaller in size than the major groove and has the benefit that it is available for attack from small molecules such as tyrosol. Most of the anticancer and antibiotic drugs that have been reported are small molecules, so the minor groove is important as their main binding site.

The stabilisation of the complex is achieved by the formation of hydrogen bond (Figure 12), polar and hydrophobic interactions. From the five hydrogen bonds between DC-11, DG-10, DG-14 and DG-16 nucleotides, three hydrogen bonds are formed between the aliphatic hydroxyl group of tyrosol and the purines of DG-10 and DG-16 base pairs. One more hydrogen bond is formed between the hydroxyl group and the pentose of DG-11 nucleotide and another hydrogen bond between the phenolic hydroxyl group of tyrosol and the purines of DG-14 base pair.

In addition, Table 8 illustrates the nucleotides, the number of hydrogen bonds, and the binding energy that are formed with tyrosol.

The above results are in accordance with the results obtained from the DNA-binding studies using UV spectroscopy, which indicated an external interaction between TYR and ctDNA.

### 3.9. DNA Binding Studies with ctDNA Using UV Spectroscopy

The interaction of the compounds and nanosystems with ctDNA was studied by UV spectroscopy to obtain information on the existence of any interaction and to further calculate the DNA-binding constants of the compounds (k_b_). An interaction between a chemical entity and DNA can disrupt the ctDNA band located at 260–280 nm in the presence of increasing amounts of ctDNA.

In absorption spectroscopy, hyperchromism and hypochromism are significant spectral features to study the changes of the double helical structure of DNA. Due to the strong interactions between a molecule and DNA bases, a change in absorption is observed, showing the proximity of the molecule to the DNA bases. On the basis of the interaction of compounds with DNA, the binding constant k_b_ for ligand–DNA binding was determined in the present work, using the Benesi–Hildebrand plot [42]. For the sake of comparison, the binding of a well-known xanthene dye, namely Rhodamine B, which has been shown to have a nonintercalative ctDNA binding in the DNA minor groove [43,44], was also studied.

It is worth noting that, in the present work, no ctDNA binding was observed for any of the nanosystems at 5 min; thus, the measurements were repeated after 30 min. This phenomenon is attributed to the nature of the nanosystems: at 5 min, no significant amount of tyrosol was released from the matrix of the nanosystem; whereas after 30 min, the released tyrosol was able to bind to ctDNA. On the other hand, the binding of rhodamine B and tyrosol to ctDNA at 5 min and 30 min did not show specific change. The UV-Vis data are summarised in Table 9.

**Figure 13:**
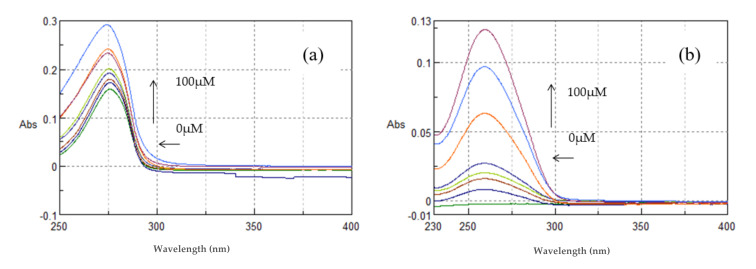
Absorption spectra of (**a**) TYR and (**b**) βCD (100 μM, pH 7.4) in Tris-HCl buffer with increasing concentrations of ctDNA (0–100 μΜ). Arrows (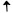) and (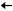) refer to hyperchromic and hypochromic (blue shift) effects, respectively.

As shown in Figure 13a, TYR (100 μM, pH = 7.4) showed absorption maxima at 275.8 nm. With incremental addition of ctDNA to the solution of TYR, an increase in the absorption intensity at 275.8 nm was observed with concomitant blue shift of the λ_max_ at 274 nm. This hyperchromism suggests that TYR binds to ctDNA by groove binding mode. The binding constant (k) of TYR was calculated from the ratio of the intercept to the slope and was found to be k = 2.09 ± 0.07 × 10^4^ M^−1^. The results indicate the binding of TYR in the minor groove of ctDNA, as predicted by the molecular modeling studies.

CDs are known to interact with nucleic acids. Recently, it was reported that when a complex with βCD is formed, the deoxyribose or ribose and the phosphate groups stabilize the docked complex by hydrogen bonds with the outer rim of the CD molecules [26,45]. In this context, the interaction of β-CD and the **TYR-β-CD** inclusion complex with ctDNA was investigated.

As shown in Figure 13b, βCD showed absorption maxima at 261 nm. With incremental addition of ctDNA to the βCD solution, an increase in the absorption intensity at 261 nm was observed with concomitant shift of the λ_max_ at 259.2 nm (blue shift). The binding constant (k) of βCD was calculated from the ratio of the intercept to the slope and was found to be k = 0.78 ± 0.05 × 10^4^ M^−1^. Therefore, it seems that βCD binds to ctDNA in a non-intercalative mode, yet much less strongly than tyrosol.

**Figure 14:**
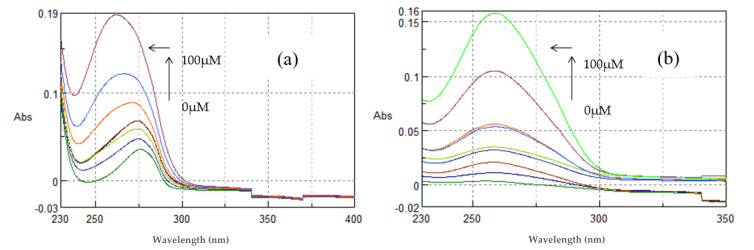
Absorption spectra of (**a**) **TYR-βCD** and (**b**) **TYR-βCD/CS** (100 μM, pH 7.4) in Tris-HCl buffer with increasing concentrations of ctDNA (0–100 μΜ). Arrows (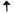) and (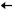) refer to hyperchromic and hypsochromic (blue shift) effects respectively.

As shown in Figure 14 the complex **TYR-βCD** showed absorption maxima at 276.4 nm. With incremental addition of ctDNA to the solution of **TYR-βCD**, an increase in the absorption intensity at 276.4 nm was observed with a blue shift at λ_max_ 272.6 nm. The binding constant was found to be k = 2.40 ± 0.16 × 10^4^ M^−1^. The complex **TYR-βCD** showed enhanced interaction with ctDNA in comparison to the free tyrosol, which could be explained by the enhanced aqueous solubility of the inclusion complex and by the mode of insertion of tyrosol in the βCD cavity. As previously mentioned, if the aromatic ring lies well inside the hydrophobic cavity, while the aliphatic hydroxyethyl group points towards the primary face of the cone, it means that hydrogen bonds can be formed and stabilize the interaction between the complex and ctDNA.

The complex **TYR-βCD/CS** showed absorption maxima at 260.2 nm (Figure 14). With incremental addition of ctDNA to the solution of **TYR-βCD/CS**, an increase in the absorption intensity at 260.2 nm was observed, with a blue shift of the λ_max_ 258.8 nm. The binding constant was found to be k = 1.30 ± 0.08 × 10^4^ M^−1^.

**Figure 15:**
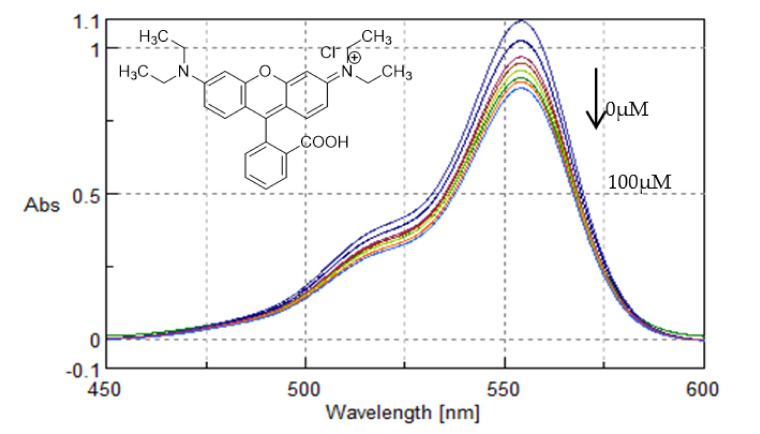
UV-Vis spectra of Rhodamine B with increasing concentrations of ctDNA (0–100 μΜ). Arrow refers to hypochromic effect.

Rhodamine B showed a hypochromic behaviour and the binding constant was determined to be 10.92 ± 0.20 × 10^4^ M^−1^ (Figure 15). This is consistent with the minor groove binding of Rhodamine B in ctDNA, in accordance with the work of Islam et al. [44].

All the tested compounds and nanosystems, with the exception of Rhodamine B, showed hyperchromism and a blue-shift upon increasing DNA concentration, indicating that they all interact with the DNA helix. However, as no significant changes in the spectra could be observed, the results indicate a nonintercalative mode of binding. CS and **TYR/CS** were also studied, but no binding with ctDNA was observed.

### 3.10. Release Profiles

**Figure 16:**
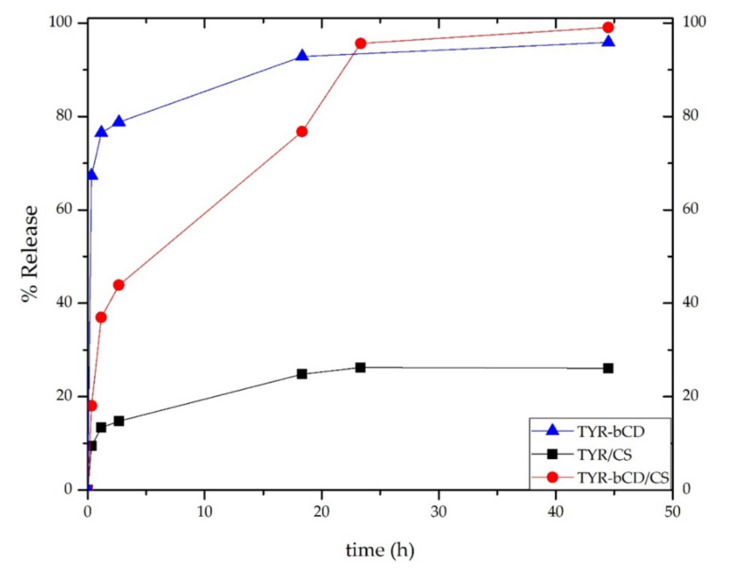
The release profile of tyrosol from **TYR-βCD** (blue line), **TYR/CS** (black line) and **TYR-βCD/CS** (red line).

The release profile of tyrosol was investigated for all nanosystems prepared, and the results are presented in Figure 16. For all systems, the release study was carried out for 45 h in a solution of pH 3.4.

All nanosystems showed a “burst release” of tyrosol during the first hour. For the **TYR-βCD** system, approximately 77% of the encapsulated tyrosol was released during that time. Thereafter, the release rate significantly slowed down, reaching 93% after 18 h.

On the other hand, the release profile of tyrosol from the **TYR/CS** system was significantly different. During the first hour of the study, 13% of tyrosol was released reaching the maximum release after 23 h (26%).

For the **TYR-βCD/CS** system, 37% of the encapsulated tyrosol was released during the first hour, reaching the 99% after 45 h. The slower rate presented by this system as compared to the **TYR-βCD** system can be attributed to the existence of chitosan as a coating and provides proof that this “double” encapsulation system can offer a more sustained release than the TYR/βCD by slowing down the initial burst effect observed in the case of the inclusion complex.

## 4. Discussion

The comparative study of the optimisation of the encapsulation parameters of tyrosol and the inclusion complex of β-cyclodextrin:tyrosol in chitosan revealed that the concentration of chitosan plays a significant role on the properties of the particles that are formed. Moreover, the ratio between the polymer and the cross-linking agent or the molecule to be encapsulated also affects the responses of the particulate system. In both systems studied, it was observed that the smaller sized particles presented a good ζ-potential, which is indicative of a very stable formulation.

The interaction of tyrosol, the inclusion complex of tyrosol with βCD and the inclusion complex coated with chitosan with ctDNA was studied in an effort to elucidate the potential of the prepared nanosystems to be exploited as pharmacologically active agents. Tyrosol and all the examined nanosystems showed a nonintercalative mode of binding to ctDNA. The complexation of tyrosol with cyclodextrin resulted in slightly better interaction with ctDNA as compared to the interaction exhibited by the **TYR-βCD/CS** system. This difference is attributed to the small, yet existent, interaction of the oligosaccharide with ctDNA whereas the polysaccharide chitosan did not interact with ctDNA in the performed experiments. Moreover, from the NMR analysis of the inclusion complex, it was deduced that the aromatic ring of the molecule lies inside the cavity, while the aliphatic hydroxyethyl moiety points towards the primary face of the cone. Therefore, it could be suggested that βCD carries the molecule close to ctDNA and an interaction can be observed. On the other hand, no interaction was observed when tyrosol was encapsulated in chitosan nanoparticles, presumably due to the strong positive charge on the surface of the **TYR/CS** nanosystem which impedes it from approaching DNA.

Finally, the release profile of tyrosol is different for each nanosystem. A burst effect is observed for all systems. The release of tyrosol from the inclusion complex formed with βCD is completed in approximately 20 h hours, but when chitosan coating is added, the release rate is delayed significantly. On the other hand, when tyrosol is encapsulated into chitosan nanoparticles, the release of only the 26% of encapsulated tyrosol occurs during the same time.

## 5. Conclusions

In the present study, the impact of two different carriers for the encapsulation of tyrosol is investigated: the oligosaccharide β-cyclodextrin and the polysaccharide chitosan. The effect of coating the tyrosol-βCD inclusion complex with chitosan was also investigated as a potential tool to modify the release profile of the bioactive compound. We were gratified to find that this was true for the systems studied in this work: the coating resulted in a sustained release of tyrosol and slowed down the initial burst effect observed from the inclusion complex.

For the formation of the tyrosol-βCD inclusion complex a well-known methodology was implemented, whereas for the two chitosan-containing systems, the formation of the nanoparticles was succeeded via the ionic gelation method with sodium tripolyphosphate. The latter processes were optimised using experimental design.

Moreover, the interaction of tyrosol and the corresponding nanosystems with ctDNA was investigated. The results show that tyrosol is a ctDNA groove binder and this was confirmed by molecular modeling studies. The same mode of binding was found for the **TYR/βCD** and **TYR/βCD/CS** nanosystems.

## Figures and Tables

**Table 1:** Factors, level and responses of the DoE studies.

Factors		Levels			Responses		Constrains
		−1	0	+1	R_1_	Size (nm)	Minimize
A	CS (% w/v)	0.05	0.20	0.35			
B	TPP (mg)	0.42	1.67	2.92	R_2_	ζ-potential (mV)	Maximize
C	(i) TYR (mg) (ii) TYR-βCD (mg)	2.5	10.0	17.5			

**Table 2:** Experimental data of **TYR/CS** nanosystem and obtained results.

No	Coded Name	Factors			Responses	
		A	B	C	R1 Size (nm)	R2 ζ-Potential (mV)
1	TYR/CS 54	1	0	−1	168.8	41.6
2	TYR/CS 52	1	−1	0	574.6	46.4
3	TYR/CS 49	−1	1	0	precipitated	4.3
4	TYR/CS 50	0	−1	−1	679.6	41.7
5	TYR/CS 77	0	0	0	127.2	24.8
6	TYR/CS 57	0	1	−1	139.4	32.0
7	TYR/CS 45	0	−1	1	474.8	36.4
8	TYR/CS 53	1	0	1	199.3	43.3
9	TYR/CS 42	0	0	0	139.8	36.4
10	TYR/CS 51	0	0	0	115.3	35.5
11	TYR/CS 56	0	1	1	148.9	37.5
12	TYR/CS 47	−1	−1	0	393.4	28.6
13	TYR/CS 43	−1	0	−1	precipitated	8.7
14	TYR/CS 46	−1	0	1	precipitated	12.7
15	TYR/CS 55	1	1	0	206.1	37.0

**Table 3:** Significance of each factor equation model terms of the TYR/CS system.

Factor	*p*-Value	
	R1	R2
Model	0.0012	0.0013
A	0.0010	0.0002
B	0.0309	0.0843
C	0.7848	0.7984
AB	0.0024	-
A^2^	0.0072	-
B^2^	0.3807	-
R^2^	0.850	0.6899
Adjusted R^2^	0.760	0.6184
Adeq Precision	11.385	10.0663

**Table 4:** Experimental data of TYR-βCD/CS nanosystem and obtained results.

Run	Coded Name	Factors			Responses	
		A	B	C	R_1_	R_2_
1	TYR-βCD/CS 67	0	−1	−1	364.8	36.9
2	TYR-βCD/CS 78	0	0	0	159.0	28.7
3	TYR-βCD/CS 66	−1	+1	0	precipitated	4.8
4	TYR-βCD/CS 73	0	+1	+1	142.2	37.5
5	TYR-βCD/CS 70	+1	0	+1	231.0	39.9
6	TYR-βCD/CS 68	0	0	0	162.5	21.2
7	TYR-βCD/CS 71	+1	0	−1	190.3	34.3
8	TYR-βCD/CS 64	−1	−1	0	276.7	19.4
9	TYR-βCD/CS 72	+1	+1	0	221.3	36.0
10	TYR-βCD/CS 69	+1	−1	0	595.0	41.9
11	TYR-βCD/CS 79	0	0	0	141.5	27.8
12	TYR-βCD/CS 62	0	−1	+1	441.5	46.5
13	TYR-βCD/CS 61	−1	0	−1	precipitated	11.7
14	TYR-βCD/CS 63	−1	0	+1	precipitated	11.3
15	TYR-βCD/CS 74	0	+1	−1	132.6	32.0

**Table 5:** Significance of each factor equation model terms of the TYR-βCD/CS system.

Factor	*p*-Value	
	R_1_	R_2_
Model	0.0028	0.0016
A	0.0006	<0.0001
B	0.1131	0.0130
C	0.7221	0.0766
AB	0.0204	0.2353
AC	-	0.3950
BC	-	0.5530
A^2^	0.0046	0.0080
B^2^	-	0.0100
C^2^	0.2881	0.0213
R^2^	0.8773	0.9760
Adjusted R^2^	0.7853	0.9327
Adeq Precision	9.8620	15.2455

**Table 6:** Chemical shift changes of ^1^H-NMR signals of βCD before and after the formation of the TYR-βCD IC.

Proton	Chemical Shifts (δ1) of βCD Protons (ppm)	Chemical Shifts (δ2) of βCD Protons (ppm) in TYR-βCD IC	Δδ = (δ2 − δ1) ppm
1	5.081	5.077	−0.004
2	3.661	3.658	−0.003
3	3.975	3.945	−0.030
4	3.596	3.595	−0.001
5	3.867–3.913	3.813	−0.100
6a,b		3.874	−0.039

**Table 7:** Characteristic FT-IR absorption bands of β-CD, tyrosol and the tyrosol-β-CD inclusion complex.

	Characteristic Absorption Bands (cm^−1^)		
	βCD	Tyrosol	Inclusion Complex
OH stretching	3382	3389	3376
C–H stretching (aromatic compounds)	-	3148	-
C–H stretching	2925	-	2924
C–H asymmetric stretching (CH_2_)	1643	-	1643
C=C stretching (Aromatic compounds)	-	1512	1541
O-H bending (alcohol)	1415	1452	1423
C–O stretching (secondary alcohols)	1029	-	1029
C–H stretching (aromatic, para substituted)	-	818	-

**Table 8:** Binding scores of the docked tyrosol on the active site of DNA.

Ligand	Binding Energy (kcal/mol)	No. of Hydrogen Bonds	Nucleotides
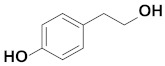	−5.0	5	DC-11, DG-10, DG-14, DG-16

**Table 9:** UV-Vis absorption data for rhodamine B, tyrosol and nanosystems in the absence and presence of ctDNA.

Compound or Nanosystem	λ_max_ Absent (nm)	λ_max_ Present (nm)	Δλ (nm)	Hypochromicity (%)	Hyperchromicity * (%)	Kb 10^4^ (M^−1^)
Rhodamine B	554	554	0	26.90	-	10.92 ± 0.20
Tyrosol	275.8	274	1.8	-	43.44	2.09 ± 0.07
βCD	261	259.2	1.8	-	93.43	0.78 ± 0.05
**TYR-βCD**	276.4	272.6	3.8	-	76.61	2.40 ± 0.16
**TYR-βCD/CS**	260.2	258.8	1.4	-	92.97	1.30 ± 0.08

* Hyperchromicity for complexes formed by compounds and nanosystems and 100 μΜ of ctDNA in comparison to free ligands.

## Data Availability

Data sharing not applicable.
